# CLIP-based multimodal endorectal ultrasound enhances prediction of neoadjuvant chemoradiotherapy response in locally advanced rectal cancer

**DOI:** 10.1371/journal.pone.0315339

**Published:** 2024-12-11

**Authors:** Hanchen Zhang, Hang Yi, Si Qin, Xiaoyin Liu, Guangjian Liu

**Affiliations:** 1 Department of Medical Ultrasonics, The Sixth Affiliated Hospital, Sun Yat-sen University, Guangzhou, China; 2 Department of Nuclear Medicine, The Sixth Affiliated Hospital, Sun Yat-sen University, Guangzhou, China; 3 Guangdong Provincial Key Laboratory of Colorectal and Pelvic Floor Diseases, The Sixth Affiliated Hospital, Sun Yat-sen University, Guangzhou, China; 4 Biomedical Innovation Center, The Sixth Affiliated Hospital, Sun Yat-sen University, Guangzhou, China; Memorial Sloan Kettering Cancer Center, UNITED STATES OF AMERICA

## Abstract

**Background:**

Forecasting the patient’s response to neoadjuvant chemoradiotherapy (nCRT) is crucial for managing locally advanced rectal cancer (LARC). This study investigates whether a predictive model using image-text features extracted from endorectal ultrasound (ERUS) via Contrastive Language-Image Pretraining (CLIP) can predict tumor regression grade (TRG) before nCRT.

**Methods:**

A retrospective analysis of 577 LARC patients who received nCRT followed by surgery was conducted from January 2018 to December 2023. ERUS scans and TRG were used to assess nCRT response, categorizing patients into good (TRG 0) and poor (TRG 1–3) responders. Image and text features were extracted using the ResNet50+RBT3 (RN50) and ViT-B/16+RoBERTa-wwm (VB16) components of the Chinese-CLIP model. LightGBM was used for model construction and comparison. A subset of 100 patients from each responder group was used to compare the CLIP method with manual radiomics methods (logistic regression, support vector machines, and random forest). SHapley Additive exPlanations (SHAP) technique was used to analyze feature contributions.

**Results:**

The RN50 and VB16 models achieved AUROC scores of 0.928 (95% CI: 0.90–0.96) and 0.900 (95% CI: 0.86–0.93), respectively, outperforming manual radiomics methods. SHAP analysis indicated that image features dominated the RN50 model, while both image and text features were significant in the VB16 model.

**Conclusions:**

The CLIP-based predictive model using ERUS image-text features and LightGBM showed potential for improving personalized treatment strategies. However, this study is limited by its retrospective design and single-center data.

## Introduction

Colorectal cancer is the third most prevalent globally, characterized by a high mortality rate [1]. The management of locally advanced rectal cancer (LARC) is based on a multidisciplinary approach. The standard therapeutic approach for LARC typically involves the use of neoadjuvant chemoradiotherapy (nCRT), followed by total mesorectal excision (TME) and subsequent adjuvant chemotherapy, aimed at tumor downsizing and downstaging [2, 3]. For newly diagnosed rectal cancers staged as cT3 or cT4 via endorectal ultrasound (ERUS) or magnetic resonance imaging (MRI), nCRT is generally advised [4]. Its benefits include tumor downstaging, reduction in local recurrence [5]. A favorable response to nCRT, such as achieving a pCR or having minimal residual tumor, is associated with improved survival outcomes [6, 7]. Those who do not respond adequately to nCRT face the dual challenge of ineffective tumor control and the adverse effects of nCRT, resulting in a poorer prognosis [8]. Thus, it is essential to develop and evaluate multiple predictive methodologies to identify LARC patients who are likely to have a favorable response to nCRT. Such approaches can help tailor treatments and support clinical decisions for individualized care.

Multiple studies have reported the predictive performance of MRI for the efficacy of nCRT in patients with LARC [9, 10]. Recent studies suggest that ultrasound may offer superior accuracy in delineating tumor boundaries and predicting tumor invasion [11, 12]. Existing evidence suggests that ultrasound has demonstrated good performance in predicting the response to nCRT in breast cancer [13–15]. If additional diagnostic information can be extracted from ultrasound images and reports to identify LARC patients who are likely to respond well to nCRT, this could guide personalized treatment decisions.

The National Comprehensive Cancer Network (NCCN) guidelines highlight the use of tumor regression grading (TRG) as a valuable tool for categorizing the response to nCRT. TRG, which assesses the degree of fibrosis relative to residual tumor cells after nCRT. It has shown great potential in predicting patients’ survival and guiding clinicians’ diagnostic and treatment decisions, ranging from TRG 0 (pathologic complete response [pCR]) to TRG 3 (poor response) [16].

Previous studies on medical imaging have predominantly relied on image data alone. For instance, Chen et al. demonstrated that radiomics features derived from both intratumoral and peritumoral regions in rectal cancer MRI can effectively predict nCRT response [17]. Similarly, Wang et al. used a machine learning model based on multiparametric MRI images to predict poor and good responders to nCRT [18]. Another study by Wang et al. showed that delta radiomics from rectal cancer MRI images can accurately predict nCRT treatment response [19]. However, ERUS medical reports typically consist of both images and structured text, and both components are essential for a complete report.

Contrastive Language-Image Pre-training (CLIP) has shown exceptional performance in various tasks by leveraging a large corpus of images and text data for pre-training [20]. By using the CLIP model, it becomes possible to fully utilize the combined image-text data. In clinical practice, integrating ultrasound images with their corresponding report text may enhance the prediction of nCRT treatment response in patients with LARC. To further explore this potential, we employ a CLIP-based model to extract features from image-text pairs, which will be used for downstream classification research.

Therefore, the objective of this study is to evaluate the efficacy of using the CLIP model to predict the TRG of LARC patients before they undergo preoperative nCRT, by leveraging ERUS images and text reports in conjunction with the CLIP model.

## Methods

### Patient selection

This retrospective study was carried out in accordance with the Declaration of Helsinki and received approval from the Ethics Committee (Approval ID 2024ZSLYEC-120). All participant information was anonymized during data collection, and no personally identifiable information was collected. The authors did not have access to information that could identify individual participants during or after data collection, which was conducted for the research purposes between June and August 2024. ERUS images—including both grayscale and Doppler images—and text reports were collected for patients diagnosed with LARC who underwent nCRT prior to surgery at our medical center between January 2018 and December 2023.The text reports contained information on the patients’ age and gender, along with detailed observations from the ERUS. This included details on the size, thickness, shape, and margins of the lesions; the internal echotexture of the lesions; their relationship to surrounding tissues; Doppler ultrasonography findings on blood flow within the lesions; the size of visible lymph nodes nearby; and a brief diagnostic summary with tumor staging. The inclusion criteria were: 1. Confirmation of LARC via ERUS, staged as uT3-4 [21] (ultrasound T stage, American Joint Committee on Cancer eighth edition). 2. Histological confirmation of LARC with post-nCRT pathology. 3. No prior treatment before nCRT. The exclusion criteria were: 1. Patients who did not receive preoperative nCRT (N = 1016). 2. Inadequate ERUS images (N = 18). 3. Incomplete clinical data (N = 13). Following the application of these criteria, a total of 577 eligible patients were enrolled.

### ERUS examination

ERUS was conducted using a Pro Focus 2202 scanner (BK Medical, Denmark) fitted with a three-dimensional endorectal transducer (8838, 6–16 MHz, BK Medical, Denmark). The patient was positioned on their left side to optimize visibility. To improve image quality, 50 mL of gel was instilled into the rectum and anal canal to dilate the rectal lumen. The transducer was then gently inserted through the rectum and advanced beyond the area of interest, enabling a thorough examination of the tumor. Following the initial assessment, detailed images of the tumor were captured. Concurrently, clinicians prepared ERUS reports based on their findings and used uT to assess the depth of tumor infiltration.

### Clinicopathologic characteristics

For patients who underwent surgery following nCRT, the tumor regression grade [22] (TRG) serves as an indicator of the tumor’s response to nCRT. Based on the TRG classification, patients were divided into two categories: those who were classified as good responders (TRG 0) and those who were categorized as poor responders (TRG 1–3).

### CLIP methodology

The analysis was conducted using the Chinese version of OpenAI’s CLIP, Chinese-CLIP [23] (https://github.com/OFA-Sys/Chinese-CLIP, version 1.5.1). Image features (grayscale and doppler) and text features were extracted using two variants of Chinese-CLIP: "ResNet50 + RBT3" (RN50) and "ViT-B/16 + RoBERTa-wwm" (VB16). Each image and report were separately extracted into 1024-length (RN50) or 512-lengh (VB16) features, which were then merged by concatenation. After balancing the samples of good and poor responders using SMOTETomek [24], we selected the features using Boruta [25] (version 0.4.3). The dataset was then split into training and testing sets in a 7:3 ratio. We employed an ensemble learning model, LightGBM [26], for classification, utilizing default model parameters. LightGBM is renowned for its excellent training speed and high accuracy. It is specifically designed to efficiently handle large-scale datasets and can process high-dimensional feature vectors with remarkable efficiency (https://lightgbm.readthedocs.io/en/stable/). The model underwent training using the training dataset and was validated using the testing dataset. The model’s performance was assessed using ROC curves (Receiver Operating Characteristic Curve), AUROC (Area Under the ROC Curve), calibration curves, and decision curve analysis. SHAP (SHapley Additive exPlanations) [27] was employed to elucidate the individual contributions of image features (IF) and text features (TF) to the model. SHAP uses Shapley values from game theory to measure the impact of each feature on model predictions. SHAP summary plots display the SHAP values for multiple samples, helping to quickly identify the most influential features and their relationships with model predictions. These plots rank features by importance, with the most significant ones at the top. The position of features in the plot provides a clear visual indication of their impact on the model’s predictions. Clinical characteristics and CLIP features are provided in S1 Table.

### Manual radiomics methodology

**Fig 1:**
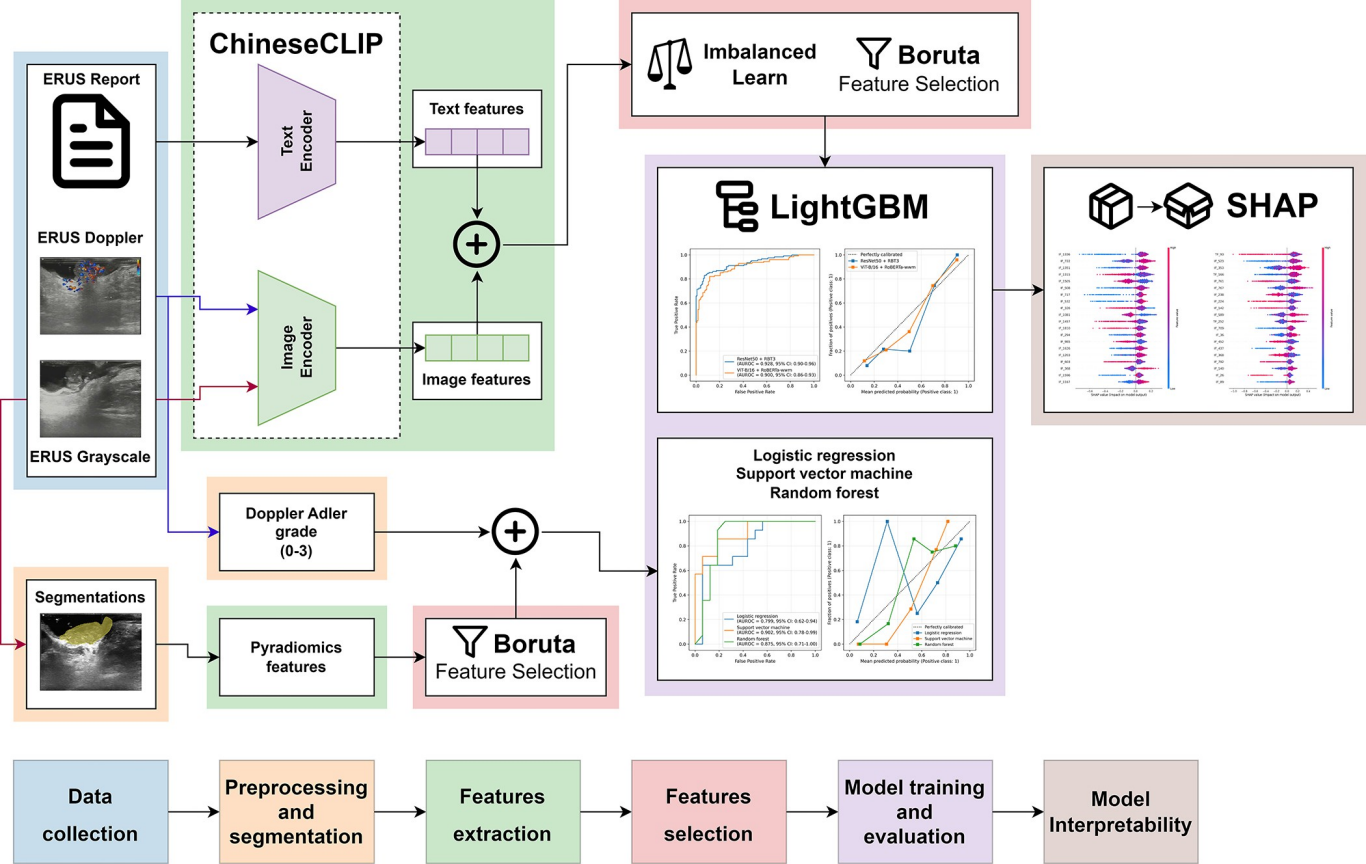
The overall research workflow.

To compare the CLIP method with the manual radiomics method, we randomly selected 100 patients (50 patients from the good responders and 50 patients from the poor responders). An ultrasonographer with three years of experience collaborated with another with eight years of experience to segment the tumor regions of interest (ROIs) in EURS images using 3D Slicer (version 5.6.2) (https://www.slicer.org/). Radiomics features including First Order Statistics, Shape-based 2D, Gray Level Co-occurrence Matrix (GLCM), Gray Level Run Length Matrix (GLRLM), Gray Level Size Zone Matrix (GLSZM), Gray Level Dependence Matrix (GLDM), and Neighboring Gray Tone Difference Matrix (NGTDM) were extracted from these ROIs using Pyradiomics (version 3.1.0) (https://pypi.org/project/pyradiomics/). For the assessment of tumor blood flow within the Doppler images, two ultrasonographers used the Adler grade [28] to score the findings. After feature selection using Boruta, the features and labels were split into training and testing sets at a 7:3 ratio. logistic regression (LR), support vector machine (SVM), and random forest (RF) models were then constructed using Scikit-learn(version 1.5.1) [29] and their performance was evaluated using ROC curves and calibration curves. The overall research workflow is illustrated in Fig 1.

### Statistical analysis

The statistical analysis was conducted using SPSS software (version 22.0; IBM Corp, Armonk, New York). Categorical variables are represented as numbers (percentages), whereas continuous variables are either summarized as median (interquartile range, IQR) or mean ± standard deviation. Data visualization was conducted using Matplotlib (version 3.9.2) [30]. A P-value below 0.05 was deemed statistically significant.

## Results

### Patient characteristics

The study included 577 LARC patients, with 28.94% being female and 71.06% male. The median age was 57.0 and the IQR was 16. ERUS uT staging revealed that 83.02%, and 16.98% of patients were at stages uT3 and uT4. 27.56% of the patients showed a positive response to nCRT, while 72.44% exhibited a poor response. The clinicopathological characteristics are summarized in Table 1.

**Table 1:** Clinicopathological characteristics.

Clinical features	All patients	CLIP Training set	CLIP Testing set	P-value	Manual Training set	Manual Testing set	P-value
	(N = 577)	(N = 652)	(N = 280)		(N = 70)	(N = 30)	
**Gender**				0.190			0.746
Female	167 (28.9%)	197 (33.7%)	82 (32.7%)		21 (30.0%)	10 (33.3%)	
Male	410 (71.1%)	388 (66.3%)	169 (67.3%)		49 (70.0%)	20 (66.7%)	
**Age**	57.0 (16.0)	56.0 (16.0)	56.0 (16.5)	0.540	56.5 (14.5)	57.0 (14.5)	0.975
**uT**				0.279			0.872
3	479 (83.0%)	506 (86.5%)	218 (86.9%)		57 (81.4%)	24 (80.0%)	
4	98 (17.0%)	79 (13.5%)	33 (13.1%)		13 (18.6%)	6 (20.0%)	
**Response**				0.475			0.667
Good	159 (27.6%)	295 (50.4%)	123 (49.0%)		34 (48.6%)	16 (53.3%)	
Poor	418 (72.4%)	290 (49.6%)	128 (51.0%)		36 (51.4%)	14 (46.7%)	
**Adler grade**							0.403
1					4 (5.71%)	6 (20.0%)	
2					16 (22.9%)	4 (13.3%)	
3					50 (71.4%)	20 (66.7%)	

### Performance of models

**Fig 2:**
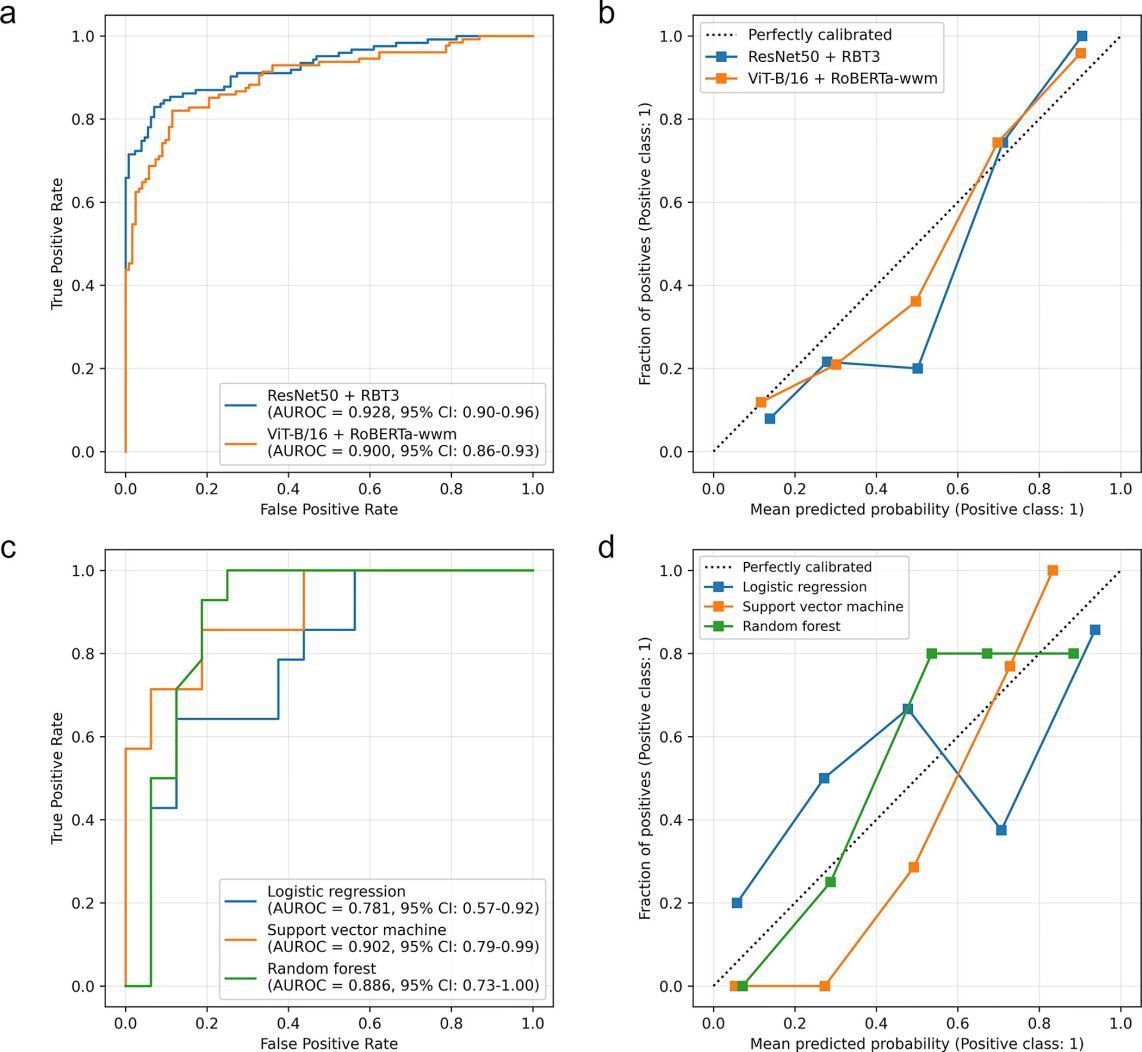
ROC curves (a, c) calibration curves (b, d) of RN50, VB16, Logistic regression, Support vector machine and Random forest on the testing set. ROC curve shows the performance of a binary classification model. It plots the true positive rate against the false positive rate at different threshold settings. AUROC summarizes the model’s performance; a higher AUROC indicates better performance.

**Fig 3:**
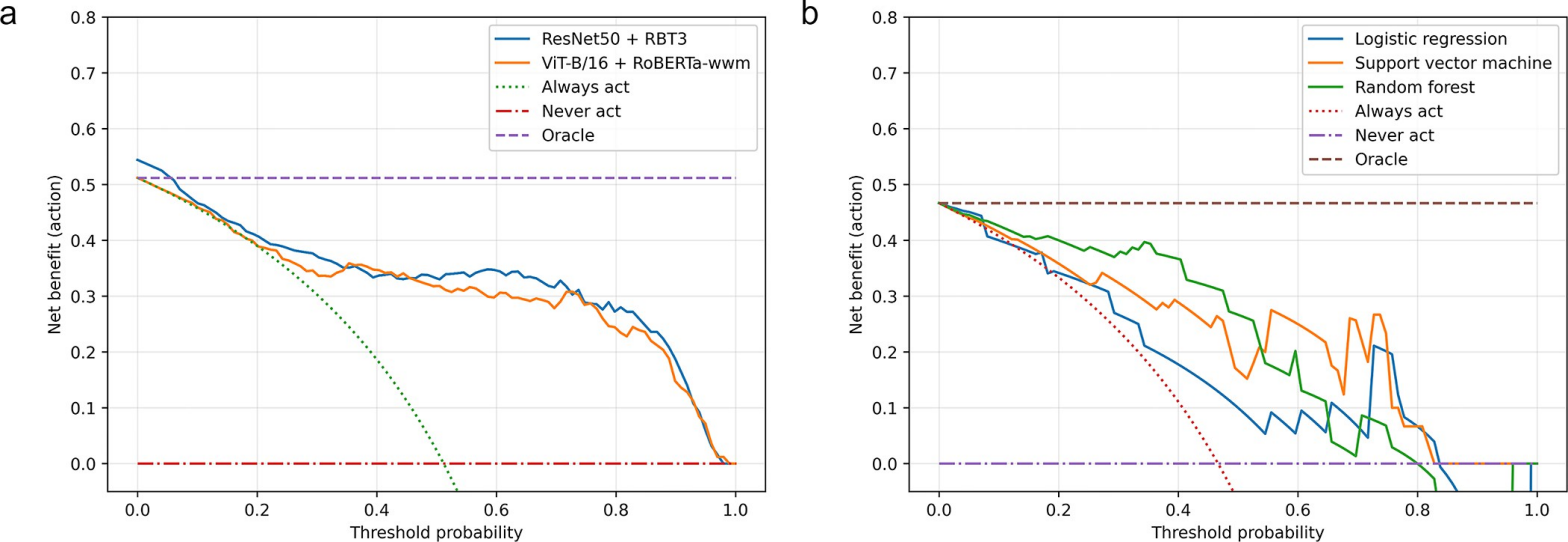
Decision curve analysis plots of CLIP models (a) and manual radiomics models (b). Decision curve analysis plot evaluates the clinical utility of a prediction model. It compares the net benefit of the model at different probability thresholds to the strategies of treating all patients or treating none. A higher net benefit indicates a more clinically useful model.

After conducting the Boruta feature selection process, we obtained 171 features for RN50 and 120 features for VB16, and 15 manual radiomics features. Fig 2 displays the ROC curves and AUROC scores for the prediction of responders in the test set. The AUROC scores for the CLIP models were comparable, with RN50 achieving a score of 0.928 (95% CI: 0.90–0.96) and VB16 achieving a score of 0.900 (95% CI: 0.86–0.93). The AUROC scores for the manual radiomics models were as follows: LR 0.781 (95% CI: 0.57–0.92), SVM 0.902 (95% CI: 0.79–0.99), and RF 0.886 (95% CI: 0.73–1.00). The AUROC score is a pivotal metric for assessing a model’s discriminative power. Our results demonstrate that CLIP-based models, exhibit higher AUROC scores in predicting nCRT responders, suggesting a greater accuracy in identifying patients who are likely to respond to treatment. This has significant implications in clinical practice, as it could enable more precise prediction of treatment responses, thereby supporting the implementation of personalized therapeutic strategies. The calibration curves (Fig 2) indicate that the RN50 and VB16 models are closer to perfect calibration than the manual radiomics models. Calibration curves serve as a measure of the consistency between a model’s predicted probabilities and the actual observed outcomes. The closer alignment of the RN50 and VB16 models to perfect calibration indicates a higher reliability in predicting the probability of response. This is crucial for clinical decision-making, as it implies that the models’ predictions can be more accurately translated into tangible clinical outcomes. The RN50 and VB16 models had higher accuracy, precision, recall, and F1-score values compared to the LR, SVM, and RF models (Table 2). DCA plots show that the net benefit for patients was generally greater with the CLIP models compared to the manual radiomics models (Fig 3). The superior performance of the RN50 and VB16 models in these metrics signifies that CLIP-based models are more effective in identifying true positives (precision) and reducing false negatives (recall), which is essential for ensuring that patients receive appropriate treatment and avoid unnecessary interventions.

**Table 2:** Comparison of model performance.

Models	Accuracy	Precision	Recall	F1-Score
**RN50**	0.86	0.88	0.88	0.88
**VB16**	0.90	0.90	0.90	0.90
**LR**	0.63	0.64	0.64	0.63
**SVM**	0.67	0.79	0.69	0.64
**RF**	0.80	0.80	0.80	0.80

### Explanations of the CLIP models

**Fig 4:**
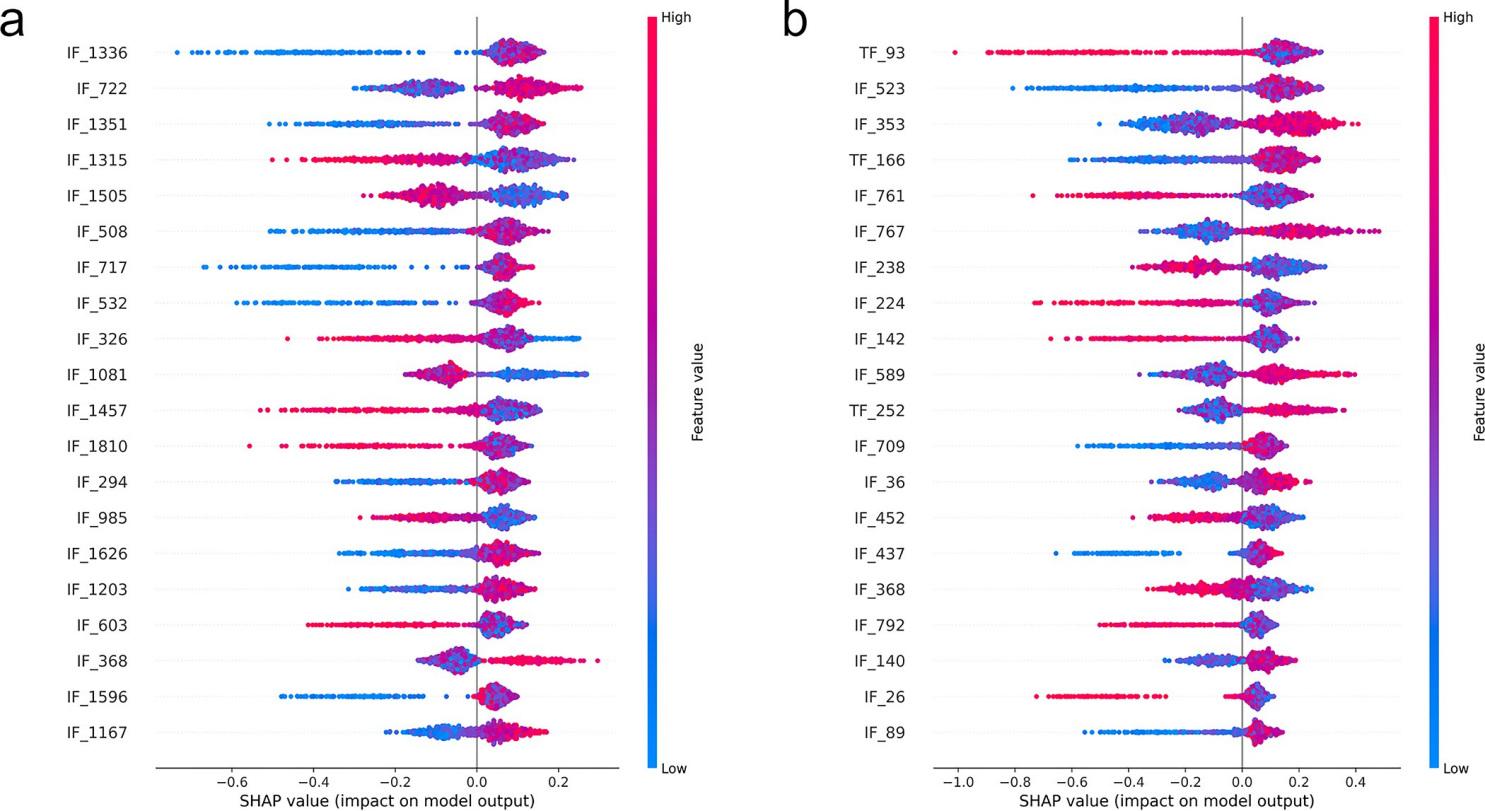
SHAP summary plots of RN50 (a) and VB16 (b). (IF: Image features. TF: Text features). SHAP summary plot shows the importance and impact of features on the model’s predictions. Each dot represents a data point, with the horizontal position indicating the SHAP value (effect on the model’s output) and the vertical position indicating the feature. Red dots represent higher feature values, while blue dots represent lower feature values. Features are ranked by importance, with the most influential at the top.

SHAP summary plots visually rank features by their influence on model predictions, highlighting the most impactful ones. This allows for a quick assessment of feature importance and their predictive relationships. The SHAP summary plot of the LightGBM model provided an importance ranking of feature variables, with the top 20 most influential variables prominently highlighted. Interestingly, both in RN50 and VB16 (Fig 4), image features dominated the top 20 list. However, text features were at the forefront in VB16. This observation indicates that both image features and text features are crucial in determining the accuracy of the models, with text features playing a notably important role in the VB16.

## Discussion

In this study, we developed effective models using CLIP features to predict responders to nCRT early. First, we collected ERUS grayscale and doppler images, and corresponding ERUS reports from patients before undergoing nCRT as our study samples. We then utilized a pre-trained Chinese-CLIP model to extract image and text features. Next, we employed these features to train a LightGBM classifier and evaluated its performance. We additionally assessed its performance in comparison to the manual radiomics approach. In the end, we utilized SHAP to examine the impact of the features on the predictions made by the CLIP model. Our results showed that the RN50 and VB16 models achieved strong predictive performance, with AUROCs of 0.928 and 0.900, respectively. These two models surpassed the LR, SVM, and RF models in terms of performance metrics. This approach offers a non-invasive and timely method for identifying poor responders to nCRT before treatment, enabling early intervention and personalized care.

From a therapeutic perspective, identifying responders before nCRT is crucial for optimizing treatment outcomes. However, relying on pathological evaluations of surgical specimens to guide treatment decisions is impractical due to the significant time delay involved. In contrast, early prediction of poor responders could enable patients to adjust their therapy plan in a timely manner.

Multiple studies have highlighted the effectiveness of different methodologies in identifying individuals who are poor responders. Chen [31] et al. evaluated the predictive potential of amide proton transfer weighted (APTw) MRI and diffusion-weighted imaging (DWI) in patients with LARC, concluding that the combined use of APTw and DWI may offer a noninvasive biomarker for assessing nCRT response. Similarly, Capelli [32] et al. assessed the predictive capacity of 18F-FDG PET/MRI in LARC patients undergoing curative-intent surgery, suggesting that PET/MRI texture analysis could serve as a valuable tool for identifying patients with a complete pathological response to nCRT. Zhou [33] et al. investigated the application of a self-attention mechanism-based multi-sequence fusion strategy in multiparametric MRI for the enhancement of nCRT response prediction in LARC. While these studies demonstrated superior predictive performance, their primary focus was on medical images rather than medical text.

CLIP, by jointly training image and text embeddings from a vast dataset, marks a seminal contribution to the contemporary field of multimodal research. We assert that our research, which synergizes CLIP with ERUS, stands as a pioneering initiative in this realm. Moreover, by utilizing a substantial sample size (N = 577), we not only bolster the credibility of our study but also facilitate a more profound understanding of the attributes of CLIP features. Wang et al. [18] demonstrated that multiparametric MRI could precisely distinguish between poor and good responders following nCRT. In contrast, our study harnessed features derived from both ERUS images and reports, rather than relying on a single modality. These multimodal features serve to complement each other, and in the specific context of ERUS, the synthesis of image and text data forms a comprehensive diagnostic outcome, potentially enhancing prognostic accuracy.

We compared RN50 and VB16 of Chinese-CLIP through a comparative analysis. Both demonstrated exceptionally accurate predictive performance, achieving AUROC scores of 0.928 and 0.900, respectively. However, VB16 demonstrated a closer alignment to the perfect calibration line in calibration curves. On the interpretative front, our analysis using SHAP revealed that RN50 predominantly leverages image-derived features for predictions, whereas VB16 incorporates a proportion of text-sourced features, potentially due to the higher parameter count of VB16’s text-side backbone, RoBERTa-wwm, compared to RN50’s RBT3. Employing VB16 or larger-scale models for the extraction of image-text features might more effectively capture textual characteristics. Chinese-CLIP [23], an open-source model trained on a vast dataset, allows researchers to meticulously examine the implementation details, thereby enhancing its reproducibility and transparency. In contrast, when we compared our CLIP method to the manual radiomics approach, we discovered that while the manual method reached a specific level of classification performance (SVM AUROC = 0.902), the manual segmentation process was highly time-consuming, labor-intensive, and usually demanded a high level of expertise. On the other hand, the CLIP method can greatly cut down the necessary time and labor, enhancing consistency.

To date, there exists no standardized definition of clinical complete response (cCR) that reliably predicts pCR. Out of the many imaging modalities available, MRI has demonstrated superior performance and accuracy. Dinapoli et al. [34] analyzed the radiomics of 221 patients across three different centers and concluded that an MRI-based radiomics model could predict pCR using pre-treatment imaging. Zhou et al. [35] analyzed multiparametric MRI data from 425 patients with LARC before nCRT, finding that specific features could effectively predict non-responders to nCRT. On the other hand, MRI is pricier and requires more time than ERUS, which has the benefit of quicker examination durations. There are very few reports on the use of ERUS for predicting nCRT response in patients with LARC. Recently, Abbaspour et al. [36] studied the predictive value of ERUS for nCRT response in LARC patients, finding that ultrasomics scores could predict pathological characteristics (AUROC = 0.83). Qin et al. [37] assessed the performance of a multi-modal ultrasomics model to predict the efficacy of nCRT, concluding that the combined model had better predictive performance(AUROC = 0.893). In contrast to these studies, our study achieved relatively better results (AUROC = 0.928) with a larger sample size. In summary, ERUS plays a significant role in assessing pCR after nCRT and can provide a stronger foundation for personalized treatment.

This study has several limitations. Firstly, it is a single-center retrospective study, which means that the model trained on data from a single institution may be biased and prone to overfitting. To address this, our next step will involve validating the model using data from multiple centers, providing a more comprehensive evaluation of its performance across diverse populations and clinical settings. While CLIP represents a novel multimodal model, it requires input data with a clear image-text pair relationship to ensure the interpretability of its output. This characteristic defines its specific scope of clinical application, highlighting both its strengths and limitations.

Future research should concentrate on the deployment of these models. There are established solutions for online deployment, such as Flask-based applications [38, 39], which would allow clinicians to input images and text reports to predict the response to nCRT in LARC patients. However, online deployment introduces several challenges, including data privacy and security, ethical and liability concerns, as well as data quality and standardization. Overcoming these challenges will necessitate collaboration among various societal sectors and clinical practitioners.

In conclusion, while our study proposes a promising approach, we acknowledge the need for further research to address the identified limitations and explore the practical application of our model in clinical settings. We look forward to contributing to the ongoing development and refinement of these models to benefit patient care.

## Conclusion

A CLIP-based model combined with LightGBM for ERUS images showed superior predictive accuracy for identifying poor responders to nCRT. This approach could significantly enhance clinical decision-making by offering more precise, patient-specific treatment strategies, ultimately improving patient outcomes and resource utilization. The integration of such models into clinical practice may lead to more efficient and personalized healthcare.

## Supporting information

S1 Table Clinical characteristics and CLIP features. (XLSX)
